# Cardiovascular Magnetic Resonance Identifies High-Risk Systemic Sclerosis Patients with Normal Echocardiograms and Provides Incremental Prognostic Value

**DOI:** 10.3390/diagnostics9040220

**Published:** 2019-12-11

**Authors:** George Markousis-Mavrogenis, Vasiliki-Kalliopi Bournia, Stylianos Panopoulos, Loukia Koutsogeorgopoulou, George Kanoupakis, Dimitrios Apostolou, Gikas Katsifis, Michail Polychroniadis, Theodoros Dimitroulas, Genovefa Kolovou, George D. Kitas, Sophie I. Mavrogeni, Petros P. Sfikakis

**Affiliations:** 1 Department of Cardiology, Onassis Cardiac Surgery Center, 17674 Athens, Greece; georgemm32@gmail.com (G.M.-M.); genovefa@kolovou.com (G.K.); 2 Joint Rheumatology Programme, National and Kapodistrian University of Athens Medical School, 15772 Athens, Greece; lily_bournia@hotmail.com (V.-K.B.); sty.panopoulos@gmail.com (S.P.); sfikakisp@gmail.com (P.P.S.); 3 Pathophysiology Department, Laikon Hospital, 11527 Athens, Greece; lukia.km@gmail.com; 4 MRI Unit, Mediterraneo Hospital, 16675 Athens, Greece; gkanoupakis@mediterraneohospital.gr (G.K.); apostolou@mediterraneohospital.gr (D.A.); 5 Department of Internal Medicine, Naval Hospital, 11521 Athens, Greece; katsifisg@yahoo.gr; 6 Department of Internal Medicine, Evangelismos Hospital, 10676 Athens, Greece; poldoc01@gmail.com; 7 Rheumatology Unit, Aristotle University of Thessaloniki, 54124 Thessaloniki, Greece; dimitroul@gmail.com; 8 Arthritis Research UK Epidemiology Unit, University of Manchester, Manchester M139PL, UK; gkitas@hygeia.gr

**Keywords:** cardiovasular magnetic resonance, systemic sclerosis, diffuse subenocardial fibrosis, myocarditis, echocardiography, adverse events

## Abstract

Background: Acute cardiac events are a significant contributor to mortality in systemic sclerosis (SSc). However, echocardiographic evaluation may be deceptively normal during an acute presentation. We hypothesized that in diffuse SSc patients presenting with acute cardiac events and a normal echocardiogram, cardiovascular magnetic resonance (CMR) would have incremental diagnostic/prognostic value. Methods. 50 consecutive diffuse SSc patients with normal echocardiograms were evaluated using a 1.5T system. A total of 27 (63%) had experienced an acute cardiac event three to tendays before CMR evaluation (rhythm disturbances, angina pectoris, shortness of breath). Left/right ventricular (LV/RV) volumes and ejection fractions (EF), as well as LV mass, the T2-signal ratio, early/late gadolinium enhancement (EGE/LGE), native/post-contrast T1-mapping, T2-mapping and extracellular volume fraction (ECV) were compared between the event and no-event groups. Results: No differences were identified in LV/RV volumes/EF/mass. In logistic regression analyses, independent predictors of belonging to the event group were EGE (odds ratio (95% CI): 1.55 (1.06–2.26), *p* = 0.024), LGE (1.81 (1.23–2.67), *p* = 0.003), T2 mapping (1.20 (1.06–1.36), *p* = 0.004) and native/post-contrast T1 mapping (1.17 (1.04–1.32), *p* = 0.007 and 0.86 (0.75–0.98), *p* = 0.025). At a median follow-up of ~1.2 years, 42% vs. 11% of the event/no-event group respectively reached a combined endpoint of event occurrence/recurrence or cardiovascular mortality. Of the independent predictors resulting from logistic regression analyses, only LGE (hazard ratio (95% CI): 1.20 (1.11–1.30), *p* < 0.001), T2-mapping (1.07 (1.01–1.14), *p* = 0.025) and native T1-mapping (1.08 (1.01–1.15), *p* = 0.017) independently predicted the combined endpoint. Conclusions: A normal echocardiogram does not preclude myocardial lesions in diffuse SSc patients, which can be detected by CMR especially in symptomatic patients.

## 1. Introduction

Systemic sclerosis (SSc) is an autoimmune disease (ARD) of unknown etiology, characterized by fibrosis of the skin and internal organs, typically microvascular vasculopathy and systemic inflammation with presence of circulating autoantibodies and pro-inflammatory cytokines [1,2]. Despite being classified as a rare disease, in actuality, SSc accounts for significant disease burden as it affects more than 2.5 million patients worldwide, with approximately 300,000 new cases being diagnosed each year [3].

SSc has an estimated 10-year survival rate of 66–82% [4,5,6,7], with cardiovascular disease (CVD) accounting for 20–30% of all deaths [8]. Microvascular disease of the myocardium can result in angina pectoris, acute myocardial infarction (MI) or both in SSc patients. Additionally, recurrent vasospasm may lead to repeated focal ischemia and consequent myocardial fibrosis [9]. Furthermore, inflammation presenting either as myocarditis [10] or as acute, diffuse subendocardial vasculopathy leading to diffuse subendocardial fibrosis [11] may also contribute to increased cardiovascular mortality.

The first non-invasive imaging modality usually employed for the cardiovascular assessment of SSc patients is echocardiography, which has the advantage of being inexpensive, fast and widely available [12]. However, it suffers from a number of drawbacks including but not limited to dependence of image quality on sufficient visual window and an inability to characterise myocardial tissues as to the presence of oedema and fibrosis [13]. These limitations are particularly pertinent in the setting of acute-onset cardiovascular symptoms/events which might not immediately be pre-empted by directly appreciable abnormalities in echocardiographic cardiac functional indices. This mismatch between pathophysiology and echocardiographic indices may lead to delays in diagnoses, and as a result thereof, also decreased survival due to lack of prompt initiation of appropriate treatment [14].

Cardiovascular magnetic resonance (CMR) is a non-invasive imaging modality that provides detailed information regarding various cardiovascular pathologies without employing ionizing radiation. CMR can make up for many of the limitations of echocardiography and is considered the reference standard for quantitative evaluation of ventricular volumes, mass, function and cardiac tissue characterization [15]. We hypothesized that CMR would have incremental diagnostic/prognostic value in SSc patients presenting with acute onset of cardiac symptoms but having a normal echocardiogram. We aimed to prospectively study a cohort of SSc patients referred to our tertiary cardiac center with de novo cardiac symptoms/events and having a normal echocardiographic evaluation on arrival, and to compare the CMR profile of these patients with SSc patients from our center with normal echocardiograms but without acute-onset symptoms.

## 2. Patients and Methods

### 2.1. Patients

We prospectively recruited 50 consecutive diffuse SSc (dSSc) patients diagnosed based on the 1980 American College of Rheumatology criteria [16]. Of these, 31 (62%) patients [the ‘event group’] had experienced one or more of the following adverse symptoms/events 3–10 days before recruitment:

- Recent-onset supraventricular arrhythmia;
- Recent-onset angina pectoris (AP);
- Sustained or non-sustained ventricular tachycardia (sVT/nsVT);
- Other ventricular rhythm disturbances (VRDs) or;
- Recent onset shortness of breath (SOB).

The remaining 19 (38%) patients experienced no such symptoms/events during that time (the ”no-event group”). All patients had undergone standardized echocardiographic evaluation [12] within one week of recruitment without identification of any abnormalities and all were evaluated using a 1.5T MR system.

### 2.2. Follow-Up, Adverse Outcomes and Clinical Endpoints

All participants were followed-up for a median duration of 1.23 (interquartile range 0.71–2.00) years after undergoing CMR evaluation and cardiovascular mortality as well as recurrence or first occurrence of adverse events as defined previously was determined. A combined endpoint was generated, which incorporated the aforementioned adverse outcomes as events.

### 2.3. Exclusion Criteria and Ethical Considerations

Exclusion criteria included contraindications to CMR, renal/liver impairment, pregnancy and hypersensitivity to gadolinium-based paramagnetic contrast agent. This study was approved by the Onassis Cardiac Surgery Center medical ethics committee (13/4/2015; identification: Mavrogeni-ARD1) and all participants provided written informed consent before inclusion.

### 2.4. Methods

Plasma levels of high-sensitivity cardiac troponin-T (hs-cTnT) were determined using sandwich enzyme-linked immunosorbent assays. CMR was performed using a 1.5-T scanner (Ingenia, Philips Medical Systems, Best, The Netherlands). The CMR protocol included standard steady-state free-precession cine CMR for the determination of left/right ventricular (LV/RV) end-systolic/end-diastolic volumes (ESV/EDV) and ejection fractions (EF), black-blood T2-weighted short tau inversion recovery images, T1-weighted spin-echo early gadolinium enhancement (EGE) CMR, and phase-sensitive inversion recovery late gadolinium enhancement (LGE) CMR as described previously [17]. A dose of 0.1 mmol/kg gadobenate dimeglumine paramagnetic contrast agents was injected for EGE and another 0.1 mmol/kg for LGE, according to the protocol suggested by the Lake Louise criteria [17].

T1-mapping was performed using a modified Look–Locker inversion recovery (MOLLI) sequence with a 3(3)5 scheme on 3 representative short-axis positions before and 15 min after contrast-medium administration. ECV was calculated from native and post contrast T1 mapping using the previously described equation [18]. T2-mapping was performed on 3 corresponding LV short axis slices using a black-blood prepared, navigator-gated, free-breathing hybrid gradient (echo planar imaging) and spin-echo multi-echo sequence [18].

### 2.5. CMR Data Analysis

Global myocardial inflammation was assessed on T2-weighted images by calculating the T2 signal intensity ratio as the ratio of the signal intensity of myocardium divided by the signal intensity of skeletal muscle [17]. Global relative enhancement was calculated by measuring myocardial signal intensity on pre- and post-contrast T1-weighted spin-echo images relative to skeletal muscle [17]. The presence and pattern of non-ischemic LGE lesions were qualitatively assessed by consensus agreement of two experienced observers. Native and post-contrast T1 maps, T2 maps and ECV were generated using dedicated plug-ins written for the OsiriX software as described previously [18]. Global native/post-contrast myocardial T1, ECV, and T2 values were calculated as the mean value of 3 short-axis slices.

### 2.6. Validation of T1 and T2 Measurements

The accuracy of the T1 and T2 mapping methods was evaluated by a relaxometry study in a Eurospin Gel-Phantom (TO5, Diagnostic Sonar LTD, Livingston, Scotland): the comparison of T1 values obtained by the MOLLI 3(3)5 and a reference scan has been previously reported [18]. T2 values obtained by the black-blood–prepared multiecho hybrid gradient and spin-echo sequence were compared with a spin-echo reference sequence with 16 echoes, 8-ms echo spacing, and 10-s time to repetition. Furthermore, myocardial T2 values were measured in 16 myocardial segments in an additional control group to assess reproducibility and regional variations of estimated myocardial T2 [18]. This control group consisted of 20 healthy subjects with a median age of 25 years (range 24–28 years) without symptoms or a history of any cardiovascular disease. In addition, inter-scan reproducibility was assessed for myocardial T1 and for T2 measurements by performing 10 repeated scans with identical imaging parameters. The interobserver reproducibility of data analysis was assessed between 2 blinded observers in all subjects [18].

### 2.7. Statistical Analysis

Statistical analyses were carried out using Stata v15.0SE. Normality of continuous variables was determined by visual assessment of histograms or Q-Q plots when necessary. Normally distributed variables are presented as mean (SD), not-normally distributed continuous variables are presented as median (interquartile range-IQR) and binary/categorical variables are presented as *n* (%). Normally distributed, continuous not-normally distributed and binary/categorical variables were compared between the event and no-event groups using independent-sample *t*-tests, Mann-Whitney U tests and chi-square tests respectively. Logistic regression analysis was used to create univariable and multivariable prediction models for discriminating between the event and no-event groups based on a backwards stepwise approach. To investigate the utility of CMR indices as predictors of the combined endpoint as defined above, univariable and multivariable Cox regression analysis was employed. Multivariable corrections for both logistic and proportional hazard models were for age, sex and disease duration (time since diagnosis at inclusion). Proportionality of hazards was determined using standardised Schoenfeld residuals. Additionally, Kaplan–Meier curves combined with the log-rank test were used for presenting the prognostic value of CMR variables visually. For the dichotomisation of predictors, we used locally established cut-off points for normal values at our center. A Benjamini–Hochberg correction for multiple comparisons was applied to account for the various logistic and Cox regression analyses that were carried out.

## 3. Results

Baseline characteristics and CMR variables are compared between the event and no-event groups in Table 1. The event group did not differ significantly from the no-event group with regard to sex (27 (87%) vs. 10 (100%) female, *p* = 0.10), age (median (IQR): 55.0 (40.0–61.0) vs. 56.0 (47.0–64.0), *p* = 0.51) or median time since diagnosis (4.0 (2.0,–8.0) vs. 3.0 (1.0–5.0), *p* = 0.44). Of the 31 patients in the event group, 17 (54.8%) experienced AP, seven (22.6%) experienced SOB, six (19.4%) experienced sVT/nsVT, three (9.7%) experienced other VRDs, and one (3.1%) experienced paroxysmal supraventricular tachycardia.

The two groups had largely similar LV and RV systolic function as well as systolic and diastolic volumes with only a trend towards significantly lower median RVEF in the event group (60.0 (50.0–65.0) vs. 65.0 (59.0–68.0), *p* = 0.065) (Table 1). No patients in the no-event group had elevated hs-cTnT, compared with nine (29%) in the event group (*p* = 0.009). Patients in the event group had noticeably higher LGE values compared to the no-event group (median (IQR): 6.0 (5.0–12.0) vs. 3.0 (0.0–5.0), *p* < 0.001) with similar differences being observed in T2-mapping (median (IQR): 63.0 (55.0–65.0) vs. 55.0 (49.00–58.0), *p* < 0.001) and native T1-mapping (median (IQR): 1135.0 (1117.0–1202.0) vs. 1065.0 (1018.0–1126.0), *p* < 0.001). Smaller but significant differences between groups were identified in T2 signal ratio, EGE, post-contrast T1-mapping and ECV (Table 1). Results of univariable and multivariable logistic regression analyses using all CMR tissue characterization indices for discriminating between the event and no-event groups are presented in Table 2. Of all indices, EGE, LGE, T2 mapping and native/post-contrast T1-mapping were independent predictors of belonging to the event group. ECV and T2 signal ratio had a trend towards significance. In multivariable stepwise backwards logistic regression analysis, each unit increase in LGE and T2 mapping predicted belonging to the event group independent of age, sex and time since diagnosis (odds ratio (95% confidence interval): 1.83 (1.22–2.75), *p* = 0.004 and 1.19 (1.03–1.38), *p* = 0.019 respectively).

**Figure 1:**
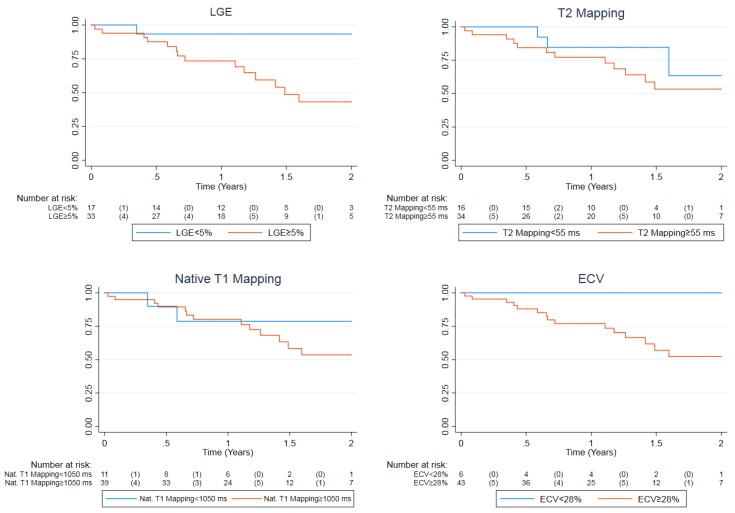
Kaplan–Meier plot for the combined endpoint demonstrating event-free survival with comparisons based on locally used normal cut-off values for LGE (<5%), T2 mapping (<55 ms), native T1 mapping (<1050 ms) and ECV (<28%). The event rates for normal vs. abnormal values were 1 vs. 14 (5.9 vs. 42.4%), 3 vs. 12 (18.8 vs. 35.3%), 2 vs. 13 (18.2 vs. 33.3%) and 0 vs. 15 (0% vs. 34.9%) respectively. The log rank test *p*-values were 0.0221, 0.302, 0.537 and 0.144 respectively. LGE, late gadolinium enhancement; ECV, extracellular volume fraction.

During a median follow-up period of 1.23 (0.71–2.00) years, 13 (41.9%) of the 31 patients in the event group reached the combined endpoint by experiencing either ventricular tachycardia (5/13), sudden cardiac death (2/13), heart failure (7/13) or pulmonary hypertension (2/13), while in the no-event group only two (10.5%) of the 19 patients experienced adverse events, those being atrial arrhythmias. Univariable Cox regression analyses demonstrated that biventricular volumes and ejection fractions, LV mass, LGE and ECV at baseline were all significant predictors of the combined endpoint (Table 3). These remained significant after multivariable correction for age, sex and disease duration. Trends towards significance were identified in univariable analyses of T2 mapping and native T1 mapping (*p* = 0.071 and 0.082 respectively). However, these became significant predictors when corrected for age, sex and disease duration (Table 3). All results remained significant after application of Benjamini–Hochberg corrections for multiple comparisons. Because LGE, T2 mapping and native T1 mapping were the only variables that could both independently discriminate between the two groups at baseline as well as being significant predictors of adverse outcomes, we generated Kaplan–Meier curves with normal cut-off values used locally at our institution. ECV was also incorporated to this selection as it was marginally non-significant in logistic regression analysis and significant in Cox regression analysis. All plots are displayed in Figure 1. Of these four, by using the locally established cut-offs of our center (LGE < 5%, T2 mapping < 55 ms, native T1 mapping < 1050 ms and ECV < 28%), LGE and ECV had the best predictive capacity for the combined endpoint. Notably, patients with a diffuse subendocardial LGE pattern had the most ominous prognosis (two experienced sudden death and two rapid evolution to heart failure). However, although the log rank test for LGE was significant (*p* = 0.022), this was not the case for ECV (*p* = 0.144).

## 4. Discussion

This study examined a cohort of SSc patients with and without an acute presentation of cardiovascular symptoms/adverse events and a normal echocardiographic evaluation according to currently employed SSc-specific protocols. At presentation, only 29% of patients in the event group had elevated hs-cTnT levels and cardiac functional indices did not differ significantly between the event and no-event groups. At a median follow-up duration of ~1.2 years, approximately 40% of the event group vs. 10% of the no-event group reached a combined endpoint of adverse event occurrence/recurrence or cardiovascular mortality. LGE, T2 mapping and native T1 mapping were the only variables that both independently discriminated between the event and no-event groups at baseline and independently predicted prognosis at follow-up.

Few studies have previously investigated SSc patients presenting with acute-onset cardiac symptoms/events and to our knowledge none have thus far incorporated CMR evaluation specifically in patients with cardiac events and normal echocardiograms. One recent study reported that myocardial fibrosis, elevated high-sensitivity c-reactive protein, and higher maximum modified Rodnan skin scores were independent predictors of cardiovascular outcomes in a cohort of 62 SSc patients presenting with cardiac symptoms [19]. However, there are serious doubts as to the validity of these findings as the statistical analyses employed in this study are either inappropriate or were performed in a false manner judging from the findings presented. In another study of 50 SSc patients with cardiac symptoms and initial screening abnormalities, 80% had one or more of the following: myocardial oedema, increased interventricular septum thickness, dilated ventricles, reduced LVEF, abnormal regional ventricular motion and/or LGE with different patterns, all without coronary distribution [20]. Additionally, a CMR-based study with two-year follow-up of 25 SSc-patients with cardiac symptoms or signs of heart failure and elevation of cardiac enzymes demonstrated that all patients who died at follow-up had a reduction of LVEF and presence of LGE at baseline [21]. A diffuse subendocardial fibrosis pattern was present in 75% of patients that died and conveyed the worse prognosis [21]. Furthermore, according to other studies, myocarditis is a common finding in SSc patients with recent-onset cardiac involvement [22,23].

To our knowledge, our study is the first in the literature providing a detailed comparison of CMR findings in dSSc patients with and without acute cardiac events. We demonstrate that ventricular function indices did not differ significantly between patients with and without events at baseline although they did have prognostic value at follow-up. In contrast, tissue characterization indices in the form of LGE, T2 mapping and native T1 mapping were the only variables that could both discriminate between the event and no-event groups at baseline and independently predict prognosis at follow-up. Considering that only 29% of patients with events had elevated hs-cTnT and all had normal echocardiograms, our findings suggest that the latter indices are important for the identification of high-risk SSc patients that may necessitate prompt initiation of treatment but may otherwise be diagnostically ambiguous. Additionally, our findings suggest that hs-cTnT testing may underestimate the presence of cardiac pathology in SSc. In spite of the diagnostic/ prognostic value of hs-cTnT measurements in coronary artery disease, the broad range of conditions associated with elevated hs-cTnT values may further confound prompt and accurate diagnosis and generate additional clinical dilemmas in SSc patients [24].

An additional innovation of our study is the evaluation of both classic and novel CMR indices. According to the modified Lake Louise criteria for inflammatory cardiomyopathies [25], a diagnosis of active acute myocarditis may be made with considerable confidence if at least one T2-based criterion (global or regional increase of myocardial T2 relaxation time or an increased signal intensity in T2-weighted CMR images) and at least one T1-based criterion (increased myocardial T1, extracellular volume, or late gadolinium enhancement) are diagnostically pathologic. At the time of writing, there are only two studies demonstrating that LGE presence on CMR (a T1-based criterion) and especially a diffuse subendocardial fibrosis pattern at baseline carries an ominous prognosis in SSc patients [21,26]. Our results are also in agreement with these studies and patients with diffuse subendocardial fibrosis from our patient cohort indeed also experienced rapid evolution to HF at follow-up. However, these studies did not include parametric indices such as T1/T2 mapping. The comparatively higher T2 mapping and native T1 mapping values in the event group emphasize the diagnostic importance of tissue characterization CMR indices in SSc next to more traditional LGE measurements. This is further reinforced by their prognostic significance in this cohort. Notably, these results are also in agreement with a previous study demonstrating that oedema is a characteristic finding in SSc patients with acute cardiac involvement [20], as T2 and native T1 mapping are sensitive to myocardial water content. In our study, EGE, post-contrast T1-mapping, and T2 signal ratio were also independent predictors of belonging to the event group. This is in agreement with the recent literature regarding the role of these parameters in the diagnosis of inflammatory cardiomyopathy [25]. However, they had no noticeable effect on prognosis in contrast to other tissue characterization indices. ECV was significant for prognosis and was marginally not-significant in discriminating between the two groups at baseline, which is most probably due to lack of statistical power.

## 5. Limitations

This study was a single-center investigation that included a moderate amount of SSc patients considering the prevalence of the disease. Although clear CMR variable cut-off points for the identification of at-risk patients would be of greater relevance for the clinician managing SSc patients, their determination was ultimately constrained due to lack of statistical power and a broad representative sample in this cohort and thus locally developed cut-off points were used. The accurate identification of such cut-off points in a larger, more diverse and representative population should be the subject of future investigations on SSc patients. Furthermore, the role of ECV in this study might have been underestimated due to a lack of statistical power. This was especially obvious for the cut-off of 28%, which perfectly identified all events at follow-up, despite ultimately not yielding a significant *p*-value. Similarly, ECV was marginally not-significant as a discriminatory parameter for distinguishing between the two groups at baseline. The same is the case for RVEF, which had a trend towards significant difference between groups at baseline.

## 6. Conclusions

This investigation demonstrates for the first time that in dSSc patients with suspected cardiac involvement and a normal echocardiographic evaluation according to current standards, only tissue characterization indices (LGE, T2 mapping and native T1 mapping) but not cardiac functional indices offer incremental diagnostic value in the identification of high risk patients at baseline investigation, in addition to independently predicting adverse outcomes at follow-up. Thus, CMR may be considered in cases where echocardiographic results are equivocal but the clinical picture warrants a higher index of suspicion, in order to identify high-risk patients early on and to institute prompt changes in the therapeutic management of SSc-related cardiac involvement.

## Figures and Tables

**Table 1:** Descriptive statistics between the event and non-event groups.

Variables	No-Event Group	Event Group	*p*-Value
**Number of Patients**	19	31	N/A
**Demographics:**			
Sex (female)	19 (100%)	27 (87%)	0.1
Age (years)	56.0 (47.0, 64.0)	55.0 (40.0, 61.0)	0.51
Years since diagnosis	3.0 (1.0, 5.0)	4.0 (2.0, 8.0)	0.44
**LV and RV Function:**			
LVEDV (mL)	124.0 (93.0, 135.0)	129.0 (112.0, 152.0)	0.16
LVESV (mL)	44.0 (30.0, 51.0)	47.0 (35.0, 76.0)	0.2
LVEF (%)	65.0 (60.0, 71.0)	63.0 (51.0, 66.0)	0.075
LV Mass (g)	61.0 (46.0, 76.0)	69.0 (58.0, 84.0)	0.13
RVEDV (mL)	110.0 (94.0, 136.0)	133.0 (100.0, 156.0)	0.16
RVESV (mL)	38.0 (31.0, 51.0)	51.0 (34.0, 69.0)	0.077
RVEF (%)	65.0 (59.0, 68.0)	60.0 (50.0, 65.0)	0.065
Elevated high-sensitivity cTnT	0 (0%)	9 (29%)	0.009 *
**Tissue Characterization Indices:**			
T2 Signal Ratio	2.2 (1.8, 2.3)	2.4 (2.0, 2.7)	0.016 *
EGE (%)	1.9 (1.4, 3.4)	3.8 (2.0, 6.0)	0.010 *
LGE (%)	3.0 (0.0, 5.0)	6.0 (5.0, 12.0)	<0.001 *
T2-Mapping (ms)	55.0 (49.0, 58.0)	63.0 (55.0, 65.0)	<0.001 *
Native T1-Mapping (ms)	1065.0 (1018.0, 1126.0)	1135.0 (1117.0, 1202.0)	<0.001 *
Post-contrast T1-Mapping (ms)	392.5 (360.0, 436.0)	340.0 (320.0, 391.0)	0.019 *
ECV (%)	30.5 (28.0, 32.0)	32.0 (31.0, 34.0)	0.009 *

LV, left ventricular; RV, right ventricular; EDV, end-diastolic volume; ESV, end systolic volume; EF, ejection fraction; cTnT, cardiac troponin T; EGE, early gadolinium enhancement; LGE, late gadolinium enhancement; ECV, extracellular volume fraction, * *p* ≤ 0.05.

**Table 2:** Results of univariable and multivariable logistic regression analysis for discriminating between the event and no-event groups using CMR tissue characterization indices. Multivariable corrections were applied for age, sex and disease duration. A Benjamini–Hochberg correction for multiple comparisons was applied on all resulting *p*-values.

Variables	Univariable		Multivariable	
	OR (95% CI)	*p*-Value	OR (95% CI)	*p*-Value
T2 Signal Ratio (per 0.2-unit change)	1.41 (1.06–1.89)	0.020 *	1.35 (0.99–1.83)	0.053
EGE (%)	1.51 (1.07–2.15)	0.020 *	1.55 (1.06–2.26)	0.024 *
LGE (%)	1.73 (1.20–2.48)	0.003 *	1.81 (1.23–2.67)	0.003 *
T2-Mapping (ms)	1.21 (1.07–1.37)	0.002 *	1.20 (1.06–1.36)	0.004 *
Native T1-Mapping (per 10 ms change)	1.19 (1.07–1.33)	0.002 *	1.17 (1.04–1.32)	0.007 *
Post-contrast T1-Mapping (per 10 ms change)	0.88 (0.79–0.98)	0.024 *	0.86 (0.75–0.98)	0.025 *
ECV (%)	1.25 (0.99–1.64)	0.051	1.22 (0.98–1.52)	0.066

CMR, cardiovascular magnetic resonance; OR, odds ratio; 95% CI, 95% confidence interval; EGE, early gadolinium enhancement; LGE, late gadolinium enhancement; ECV, extracellular volume fraction, * *p* ≤ 0.05.

**Table 3:** Results of univariable and multivariable Cox regression analysis for the combined endpoint at two years of follow-up. Multivariable corrections were applied for age, sex and disease duration. A Benjamini–Hochberg correction for multiple comparisons was applied on all resulting *p*-values. * *p* ≤ 0.05.

Variables	Univariable		Multivariable	
	HR (95% CI)	*p*-Value	HR (95% CI)	*p*-Value
LVEDV (per 5 mL change)	1.05 (0.95–1.16)	0.365	1.05 (0.95–1.17)	0.322
LVESV (per 5 mL change)	1.24 (1.08–1.42)	0.002 *	1.25 (1.08–1.45)	0.003 *
LVEF (per 5% change)	0.62 (0.49–0.79)	<0.001 *	0.63 (0.49–0.81)	<0.001 *
LV Mass (per 5 g change)	1.11 (1.01–1.24)	0.039 *	1.11 (1.01–1.23)	0.036 *
RVEDV (per 5 mL change)	1.05 (1.01–1.09)	0.037 *	1.05 (1.01–1.09)	0.029 *
RVESV (per 5 mL change)	1.08 (1.02–1.14)	0.005 *	1.09 (1.03–1.15)	0.003 *
RVEF (per 5% change)	0.70 (0.55–0.90)	0.006 *	0.68 (0.53–0.88)	0.003 *
T2 Signal Ratio (per 0.2-unit change)	1.05 (0.85–1.30)	0.651	1.12 (0.89–1.42)	0.341
EGE (%)	1.09 (0.95–1.24)	0.226	1.11 (0.97–1.27)	0.140
LGE (%)	1.16 (1.09–1.24)	<0.001 *	1.20 (1.11–1.30)	<0.001 *
T2-Mapping (ms)	1.05 (0.99–1.11)	0.071	1.07 (1.01–1.14)	0.025 *
Native T1-Mapping (per 10 ms change)	1.06 (0.99–1.12)	0.082	1.08 (1.01–1.15)	0.017 *
Post-contrast T1-Mapping (per 10 ms change)	0.96 (0.89–1.04)	0.366	0.93 (0.84–1.03)	0.182
ECV (%)	1.19 (1.05–1.35)	0.006 *	1.18 (1.04–1.34)	0.011 *

HR, hazard ratio; 95% CI, 95% confidence interval; LV, left ventricular; RV, right ventricular; EDV, end-diastolic volume; ESV, end systolic volume; EF, ejection fraction; EGE, early gadolinium enhancement; LGE, late gadolinium enhancement; ECV, extracellular volume fraction, * *p* ≤ 0.05.
